# Determinants of Dietary Vitamin D Intake in Population-Based Cohort Sample of Polish Female Adolescents

**DOI:** 10.3390/ijerph191912184

**Published:** 2022-09-26

**Authors:** Katarzyna Lachowicz, Małgorzata Stachoń

**Affiliations:** Department of Dietetics, Institute of Human Nutrition Sciences, Warsaw University of Life Sciences (SGGW-WULS), 159c Nowoursynowska Street, 02-776 Warsaw, Poland

**Keywords:** vitamin D, food products, fish, fish products, female adolescents, Poland

## Abstract

Vitamin D has a pleiotropic effect and its deficiency is a risk factor for many diseases. The purpose of this study was to analyze the dietary intake of vitamin D and the factors determining this intake by female post-primary school students in Poland. The study was conducted on a nationwide sample of 4469 female Polish adolescents (aged 14–20) recruited from all regions across Poland. The vitamin D intake was assessed using VIDEO-FFQ (Vitamin D Estimation Only—Food Frequency Questionnaire). The median dietary vitamin D intake was 2.33 μg per day and it was lower than the 15 μg recommended in Poland for more than 98% of the group. The highest vitamin D intake per day was noted for fish (0.52 μg), whereas the lowest was noted for fats (0.04 μg). Factors that influenced the dietary vitamin D intake were the amount and species of fish consumed, region of residence, use of vitamin D supplements, and vegetarian or vegan diets. In contrast, vitamin D intake did not depend on body mass index and age. Based on the results of the survey, it can be concluded that the alarmingly low intake of vitamin D by Polish female adolescents is a result of the limited supply of vitamin D primarily from fish and fish products. This may be due to insufficient nutritional knowledge, indicating an urgent need to educate the surveyed population in this area.

## 1. Introduction

Vitamin D, as a micronutrient and steroid hormone [1], has a multidirectional (pleiotropic) effect [2]. The nuclear receptor for calcitriol (VDR) is responsible for the biological activity of vitamin D and is present in the cells of many tissues [3,4] to mediate the regulation of approximately 3% (over 1000) of human genes [5,6]. In addition to the calcemic effect (related to the regulation of bone metabolism and calcium-phosphate homeostasis) [7], the active form of vitamin D has anti-inflammatory and immunomodulatory effects [8,9,10] and plays a key role in the regulation of cell differentiation and proliferation [11] and blood pressure. It is also important for the functioning of the heart and kidneys [12,13], the nervous [14,15], and digestive [16] systems, as well as the female and male reproductive systems [17]. The results of many in vivo and in vitro studies indicate that vitamin D deficiency is also associated with an increased risk for respiratory diseases [18], or certain cancers [19]. Obesity [20] and other metabolic syndrome components [21], type 2 diabetes mellitus [22], sarcopenia [23], polycystic ovary syndrome and other gynecological and obstetric diseases [17], and depression [24] are also disorders related to vitamin D deficiency.

Vitamin D comes from three sources: (1) skin production (80–90%) from pro-vitamin D3 (7-dehydrocholesterol) when exposed to sufficient ultraviolet B (UVB) radiation, (2) food intake, and (3) supplements. Vitamin D is naturally found as cholecalciferol (vitamin D3) in fatty fish (mackerel, salmon, tuna, herring, and rainbow trout), eggs, and liver, or as ergocalciferol (vitamin D2) in edible fungus [25]. The best indicator of vitamin D status available is 25-hydroxyvitamin D (25(OH)D), which is formed after hydroxylation of D3 or D2 (absorbed from the gastrointestinal tract) in the liver. In the kidney, 25(OH)D is metabolized to the more active form i.e., 1,25-dihydroxyvitamin D (1,25 (OH)2D), the level of which is not used as the indicator of vitamin D status, as it is regulated by parathyroid hormone (PTH), calcium, phosphate, fibroblast growth factor 2 (FGF-2), and 1,25(OH)2D itself (renal negative feedback loop) [25,26].

Although vitamin D is essential not only for bone health but also for extra-bone health, its deficiency at every age and in each gender, ethnic and socioeconomic group is a major public health problem worldwide [27,28]. The high prevalence of low vitamin D intake and vitamin D deficiency or inadequate vitamin D status in children and adults in Europe remains widespread [28,29,30,31]. Data from Europe showed that the prevalence of vitamin D deficiency (defined as 25(OH)D3 levels below 20 ng/mL or 50 nmol/L) and inadequacy (defined as 25(OH)D3 below 12 ng/mL or 30 nmol/L) was in 40.4% and 13.0% of the general population, respectively [28,30]. Vitamin D deficiency (defined as above) was found in <20% of the population in Northern Europe and 30–60% in Western, Southern, and Eastern Europe. Severe vitamin D deficiency (defined as above) was observed in >10% of Europeans [31]. In Poland, 90% of adults and adolescents were shown to suffer from vitamin D deficiency of varying severity [32].

Global public health interventions to avoid severe vitamin D deficiency mainly involve vitamin D supplementation and food fortification [33]. Both vitamin D2 and vitamin D3 are used in fortified foods and supplements. Vitamin D is added to milk, yogurt, cheeses, butter, margarine, orange juice, soy beverages, and other vegetable origin beverages (such as almond and oat), and breakfast cereals [25]. The fortification of these products does not apply to Poland. With the exception of a few products, the other fortified products mentioned are rarely found in the market [34,35]. Diet, including consumption of natural sources of vitamin D, such as oil-rich fish, can also be an important factor influencing vitamin D status in at-risk groups. This is particularly important in the absence of sufficient availability/exposure to sunlight to allow synthesis in the skin [28], as occurred during the COVID-19 pandemic restrictions [36]. Home confinement has led to lower serum vitamin D levels among the pediatric population in Poland (1 month to 18 years) [37] and increased the percentage of children and adolescents with vitamin D deficiency [37].

Given the high prevalence of vitamin D deficiency in adolescents [28,32] and insufficient exposure to UV radiation [28], dietary vitamin D intake should always be an important aspect to assess in adolescents, especially females.

Therefore, the purpose of this study was to analyze the dietary intake of vitamin D, its sources, and the determinants in female post-primary school students in Poland.

## 2. Materials and Methods

### 2.1. Study Population

The study was conducted on a nationwide sample of Polish post-primary school students between 1 February 2022 and 31 March 2022. The following types of post-primary schools were included: stage I sectoral vocational, general secondary, and technical secondary schools. The study was carried out in accordance with the guidelines of the Declaration of Helsinki and approved by the Ethics Committee of the Central Clinical Hospital of the Ministry of Internal Affairs and Administration in Warsaw (No. 2/2021).

Poland is divided into six main geographic regions with sixteen basic administrative units (voivodeships), depending on historic, cultural, economic, and geographic factors. Additionally, each voivodeship is further divided into counties. The participants were recruited based on a stratified random sampling of schools to obtain a representative sample of all the regions of Poland: the Central region (in which the Łódź and Mazovian voivodeships are located), the Eastern region (in which the Holy Cross, Lublin, Podlaskie and Subcarpathian voivodeships are located), the Northern region (in which the Kuyavian-Pomeranian, Pomeranian and Warmian-Masurian voivodeships are located), the North-Western region (in which the Greater Poland, Lubusz and West Pomeranian voivodeships are located), the Southern region (in which the Lesser Poland and Silesian voivodeships are located) and the South-Western region (in which the Lower Silesian and Opole voivodeships are located). The sampling procedure used in this study is in agreement with those used in other studies conducted in Poland [38].

Sampling was conducted in two phases: schools were sampled from counties, and counties were sampled from voivodeships. Firstly, the stratified sampling of schools was conducted for all regions, that is, from each voivodeship of the country 10 counties were randomly sampled (resulting in 160 counties). Secondly, 10 post-primary schools were randomly sampled from each county (resulting in 1600 post-primary schools). If there were not enough schools in the drawn county (10) then the missing schools were drawn from neighboring counties.

All the 1600 post-primary schools were invited to voluntarily participate in the study, by contacting their principals. The principals were informed of the purpose and protocol of the study and were also given an electronic link to the dedicated questionnaire. If the principals agreed to allow the school’s students to take part in the survey, they were asked to provide an electronic version of the questionnaire to the students. Students’ participation was also voluntary. All participants’ parents or legal guardians provided informed consent for their participation in the study. The questionnaire form was anonymized and did not allow the gathering of any data that would allow for the identification of the respondent. The students were informed of the purpose and objectives of the survey, and principals or their designated teachers supervised the survey.

The questionnaire was completed and returned by 7947 post-primary school students. The following criteria for inclusion in the study were adopted: being a student of a school whose principal agreed to participate in the study and age of 14–20 years (a typical age for this level of education in Poland). The following criteria for exclusion from the study were adopted: males, pregnancy or lactation, incomplete questionnaire, unreliable data included in the questionnaire (concerning, for example, body weight and/or height, number of servings of consumed products and/or product groups). After taking into account the above inclusion and exclusion criteria, the study group included 4469 female post-primary school students from all voivodeships in Poland.

**Figure 1:**
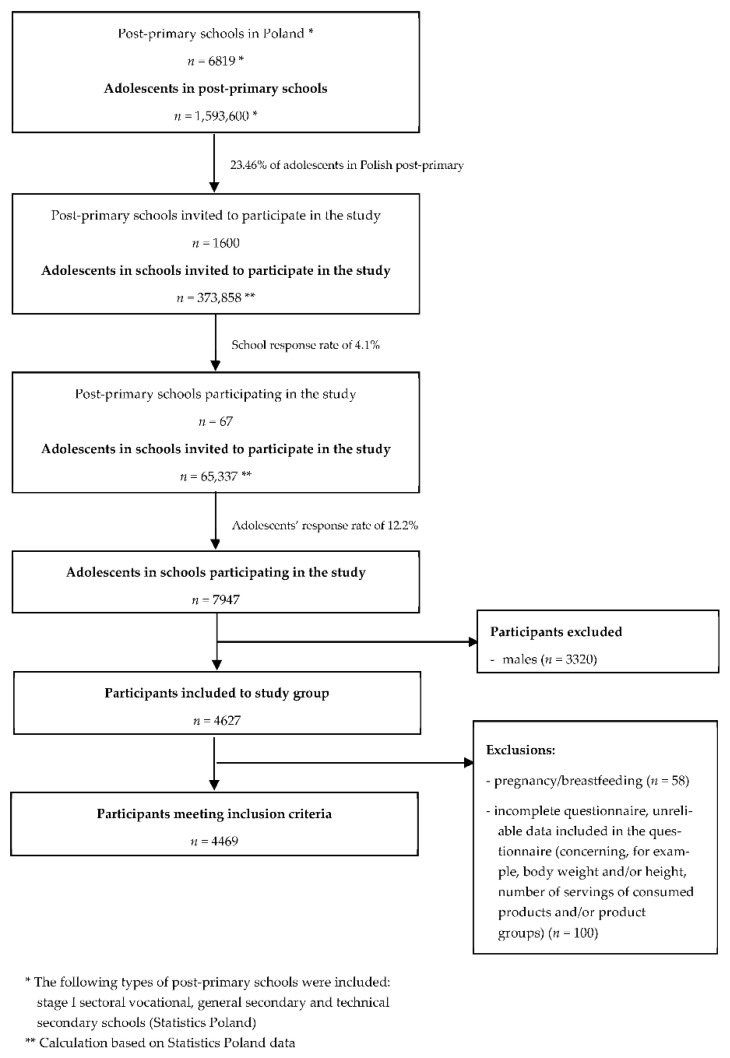
The sampling and recruitment procedure for the study group.

The sampling and recruitment procedure for the study group is shown in Figure 1.

### 2.2. Applied Questionnaire

All data for analysis in this survey were collected using the Computer-Assisted Web Interview (CAWI) method [39].

The questionnaire used in this study included questions to verify the inclusion and exclusion criteria, namely questions about gender, age, and the name of the school in a particular voivodeship and county.

In addition, the questionnaire included questions about the use of vitamin D supplements and the current use of a vegetarian (or vegan) diet (evaluated on the basis of self-declaration—no or yes).

The brief Food Frequency Questionnaire (FFQ) to assess vitamin D intake from food previously validated in a group of Polish young women (Vitamin D Estimation Only—Food Frequency Questionnaire (VIDEO-FFQ)) [40] was the relevant part of the questionnaire used in the present study. VIDEO-FFQ is a quick and convenient tool of high validity and reproducibility, which enables assessing vitamin D intake from food in large populations. It is included in the Register of Validated Short Dietary Assessment Instruments by the National Institutes of Health (NIH)—National Cancer Institute of the United States of America [41]. The applied VIDEO-FFQ included questions about the intake of specified food products during the year before the survey, independent of season, while respondents were asked to specify the number of servings per month or week, depending on the product (open-ended questions). Respondents were asked to specify the number of products consumed and those added to dishes, while they were allowed to indicate the number of servings not only as integers but also as decimal parts of servings [40]. Based on the collected data, the usual daily intake of vitamin D from food was calculated for each respondent, using formulas developed for the VIDEO-FFQ questionnaire, based on the Polish Tables of Food Composition [42]. The procedure applied allowed calculation of total vitamin D intake from the diet, as well as vitamin D intake from specific groups of products, such as (1) fish and fish products (1.1. salmon, rainbow trout, herring, eel; 1.2. halibut, mackerel, brook trout, sole, tuna; 1.3. cod, flounder, plaice, pollock, hake; 1.4. herring, sardine, and tuna products; 1.5. other fish products); (2) dairy products; (3) eggs; (4) meat and meat products; (5) cereals and (6) fats [40].

A cut-off point of 15 µg was chosen to compare the data obtained with the Polish recommendations for daily vitamin D intake, as recommended for girls aged 10–18 years and women aged 19–30 years [43]. Quartiles (Q) of vitamin D intake from food (μg) were calculated on the basis of total daily vitamin D intake from six product groups (fish and fish products; dairy products; eggs; meat and meat products; cereals and fats)—Q1 (0.01–1.32 μg), Q2 (1.33–2.32 μg), Q3 (2.33–4.03 μg) and Q4 (4.04–29.34 μg).

BMI (Body Mass Index) was calculated from self-reported weight and height using the Quetelet equation (weight (kg)/height^2^ (m^2^)). BMI for minors (14–18 years old) was assessed using the OLAF program (“Elaboration of the reference range of arterial blood pressure for the population of children and adolescents in Poland—PL0080 OLAF”) [44] based on Polish height reference curves, which are gender and age-specific [45], while BMI for adults (19–20 years old) was assessed using the World Health Organization classification [46].

### 2.3. Statistical Analysis

The Shapiro–Wilk test verified the normality of the distribution of the obtained data. Due to nonparametric distributions Chi-squared test for comparisons of proportions, the Mann–Whitney U test and Kruskal–Wallis analysis of variance (ANOVA) to compare subgroups, and the Spearman rank coefficient to analyze correlations were used. Statistical analysis was performed using Statistica, version 13.3 (TIBCO Software Inc., USA). A level of significance was chosen as *p* ≤ 0.05.

## 3. Results

### 3.1. Study Group Characteristics

The general characteristics of the study group (distributed by regions of Poland, age, BMI classification, vitamin D supplementation, and vegetarian or vegan diet followed) are presented in Table 1. In addition, the table shows the distribution of participants by quartiles of vitamin D intake.

The highest percentage of female post-primary school students came from the Northern region of Poland (30.2%), followed by the Southern (22.2%) and Eastern (20.4%) regions, while the lowest percentage came from the South-Western region (5.6%). Most of the participants were 14–17 years old (70.06%), and the remaining 29.94% were of legal age (18–20 years). Most of the participants in the study were of normal weight (72.4%) and did not follow a vegetarian or vegan diet (92.0%). A similar percentage of respondents supplemented (50.3%) and did not supplement vitamin D (49.7%).

The number of participants in subgroups differentiated by quartile of vitamin D intake depended on the region of Poland, vitamin D supplementation, and use of a vegetarian or vegan diet. In contrast, BMI and age did not affect the variation in the number of female students divided according to the quartile of vitamin D intake (Table 1).

### 3.2. Dietary Vitamin D Intake and Its Sources

**Figure 2:**
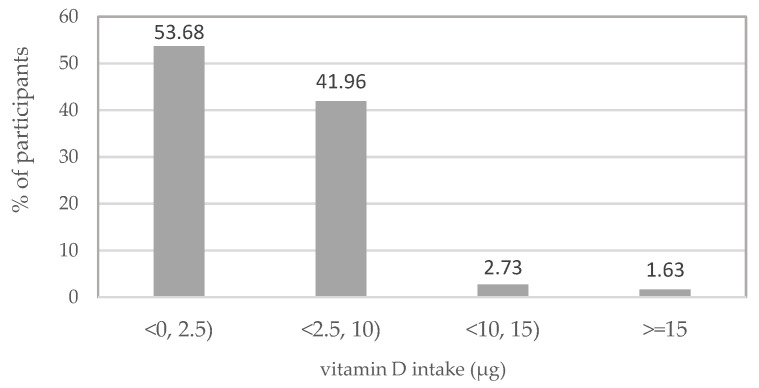
Percentage of female post-primary school students consuming vitamin D per day in ranges: <0, 2.5), <2.5, 10), <10, 15) and ≥15 µg.

The median dietary intake of vitamin D in the studied group of Polish female post-primary school students was 2.33 μg per day. Vitamin D consumption ranged from 0.01 μg to 29.34 μg per day (Table 2). It was lower than 15 μg per day recommended in Poland [42] for more than 98% of female students. Additionally, about 96% of participants had a vitamin D intake lower than 10 µg, and 54% had a vitamin D intake lower than 2.5 µg (Figure 2). A quarter of the participants consumed no more than 1.32 μg of vitamin D (Q1), while three-quarters consumed no more than 4.03 μg per day from food sources (Q3) (Table 2).

Vitamin D intake from various food groups (including different species of fish and fish products) in the studied group of female post-primary school students is presented in Table 2.

The highest median dietary vitamin D intake was noted for fish (0.52 μg per day), meat and meat products (0.39 μg per day), eggs (0.38 μg per day), and dairy products (0.31 μg per day), whereas the lowest for cereals (0.12 μg per day) and fats (0.04 μg per day). Dietary vitamin D intake ranged from 0.00 μg to 27.28 μg for fish, 8.71 μg for eggs, 4.33 μg for meat and meat products, 2.48 μg for cereals, and 3.20 μg for fats. The highest median of vitamin D intake from different species of fish and fish products was reported for salmon, rainbow trout, herring, and eel, whereas the lowest was for the other group of fresh or smoked fish, herring, sardine and tuna products, and other fish products.

The number of servings of fish and fish products consumed per week, as declared by participants, is shown in Table 3.

The median intake of total fish and fish products amounted to 0.70 servings per week, ranging from 0.00 to 34.53 servings per week. The highest number of servings was declared for salmon, rainbow trout, herring, and eel (0.23 servings per week), whereas the lower for the other groups of fish, herring, sardine and tuna products, and other fish products.

Both the median vitamin D intake of the six food groups and the median number of servings of fish and the other food groups analyzed were lowest in the first quartile subgroup and highest in the fourth quartile subgroup (details in Table 2 and Table 3).

**Figure 3:**
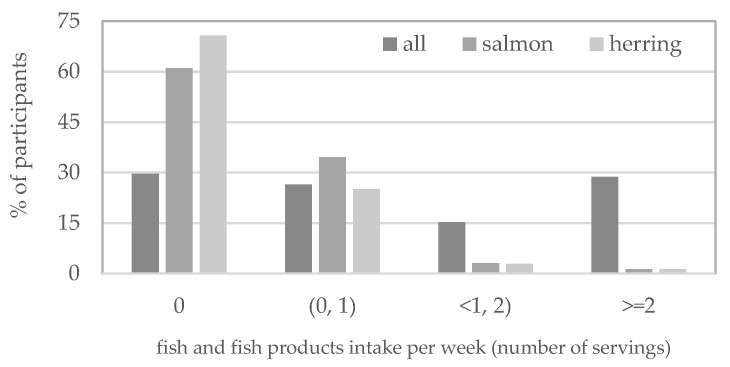
Percentage of female post-primary school students not consuming and consuming (0, 1), <1, 2) and ≥2 servings of fish and fish products including salmon and herring.

Nearly 30% of the study’s participants did not eat fish at all or consumed a minimum of two servings of fish per week, 26.43% no more than one serving, and 15.26% minimum of one serving, but no more than two servings (Figure 3).

Salmon and herring were consumed by 39% and 29.22% of the female students surveyed, respectively (Figure 3), and these fish were the source of the highest amount of vitamin D in the diet (median intake was 0.23 µg and 0.18 µg per day, respectively—Table 2). The median weekly number of servings consumed was 0.23 for salmon and 0.21 for herring (Table 3).

Both the range of vitamin D intake from salmon and herring and the range of the number of servings of these fish were lowest in the first quartile subgroup and highest in the fourth quartile subgroup (details in Table 2 and Table 3).

### 3.3. Dietary Vitamin D Intake in Subgroups

#### 3.3.1. Regions of Poland and Vitamin D Intake

Table 4 presents the comparison of dietary vitamin D intake including its consumption from various sources in the subgroups of female post-primary school students from different regions of Poland.

Participants from Northern Poland were found to have higher total vitamin D intake from food than those from Eastern Poland (*p* = 0.0008). Dietary intake of vitamin D was not significantly different between subgroups from the other regions.

Vitamin D intake from fish and fish products was higher in the respondents from Southern Poland than in the ones from Northern Poland (*p* = 0.0329). Its intake from halibut, mackerel, brook trout, sole, and tuna differed in participants from Southern and North-Western Poland, and Southern and North Poland (*p* < 0.0001). In addition, the range of vitamin D intake from herring, sardine, and tuna products was higher in female students from the Southern region than in the Northern region (*p* < 0.0001). Intakes of vitamin D from eggs, cereals, and meat and meat products were lower in Eastern Poland than in other regions (*p* < 0.0001). Furthermore, respondents from the Northern region of Poland were characterized by a higher intake of vitamin D from meat and meat products than the students living in the Central and Southern regions of Poland (*p* < 0.0001). Higher vitamin D intake from fats was observed in adolescents from Southern, South-Western, and Northern regions than in the rest of Poland (*p* < 0.001).

There were no differences in vitamin D intake from dairy products and the following groups of fish: salmon, rainbow trout, herring, and eel, as well as cod, flounder, plaice, pollock and hake, and other fish products between regions of Poland.

#### 3.3.2. Age and Vitamin D Intake

The comparison of dietary vitamin D intake including its consumption from various sources in the age subgroups is presented in Table 5.

Age did not affect the total intake of vitamin D, as well as its basic food sources (fish and their products, eggs, meat, and dairy). In contrast, vitamin D intakes from cereals and fats were higher in older participants.

#### 3.3.3. BMI Classification and Vitamin D Intake

The comparison of dietary vitamin D intake including its consumption from various sources in the subgroups of underweight, normal weight, overweight and obese female post-primary school students is presented in Table 6.

There were no differences in total vitamin D intake and its intake from six groups of food products and various fish species between subgroups of underweight, normal weight, overweight, and obese female participants.

#### 3.3.4. Vitamin D Supplementation and Vitamin D Intake

The comparison of dietary vitamin D intake, including its consumption from various sources in the subgroups of non-supplementing and vitamin D-supplementing female post-primary school students, is presented in Table 7.

It was found that participants supplementing vitamin D had a higher total intake of vitamin D from food than non-supplementing subjects (*p* < 0.0001). In addition, vitamin D intakes from fish and fish products, dairy products and eggs were higher in vitamin D-supplemented subjects (*p* < 0.0001, *p* = 0.0002 and *p* = 0.0026, respectively). There were no differences in vitamin D intake from cereals and meat and meat products and fats between vitamin D-supplementing and non-supplementing subgroups.

Vitamin D intakes from the following fish groups: salmon, rainbow trout, herring, and eel, as well as halibut, mackerel, brook trout, sole, and tuna were also higher in the supplementation subgroup (*p* = 0.0001 and *p* < 0.0001, respectively). In addition, vitamin D intake from cod, flounder, plaice, pollock, and hake differed between non-supplementing and supplementing participants (*p* = 0.0002). There were no differences in vitamin D intake from herring, sardine and tuna products, and other fish products between vitamin D-supplementing and non-supplementing subgroups.

#### 3.3.5. Vegetarian or Vegan Diet and Vitamin D Intake

The comparison of vitamin D intake from different sources in subgroups of female post-primary school students following an omnivorous vs. vegetarian or vegan diet is presented in Table 7.

It was found that participants following an omnivorous diet had a higher total intake of vitamin D from food than vegetarians or vegans (*p* < 0.0001) as well as vitamin D intake from fish and fish products and fats (*p* < 0.0001 and *p* = 0.0007, respectively). Vitamin D intakes from all various groups of fish and fish products were higher in participants following an omnivorous diet. Meat and meat products were not a source of vitamin D for vegetarians or vegans.

There were no differences in vitamin D intake from dairy products and eggs, but their percentage of daily vitamin D intake was higher in vegetarians or vegans (28.4% and 28.2%, respectively) than in participants following an omnivorous diet (17.7% and 20.3%, respectively).

### 3.4. Correlations

The results of the analysis of the correlation between the declared number of servings of fish and fish products per week and vitamin D intake from them and BMI and total dietary vitamin D intake in the group of female post-primary school students are presented in Table 8.

The analysis showed a statistically significant positive correlation between the intake of fish and fish products and the intake of vitamin D from these sources. The strongest relationship was found for salmon, rainbow trout, herring, and eel.

Significant but weak positive correlations were observed between total vitamin D intake and BMI.

## 4. Discussion

The present study evaluated the vitamin D intake from various sources in the population of Polish female adolescents and found that the average intake of vitamin D was very low (2.33 µg per day) and much lower than the recommendations. Additionally, this study showed that the intake of vitamin D is particularly lower in female adolescents from specific regions of Poland and much lower in those who do not supplement vitamin D, as well as vegetarians and vegans.

Widespread vitamin D deficiency in Poland is a serious public health problem, and the youth population is a high-risk group for low vitamin D status [47,48]. Due to unfavorable lifestyle changes, as well as a low supply of vitamin D from food and limited cutaneous synthesis, the situation may worsen. Therefore, it is essential to take appropriate preventive and intervention measures. One such activity was the updating of guidelines for vitamin D supplementation in the general population and groups at risk of vitamin D deficiency in Poland by national consultants and representatives of Polish and international scientific organizations/societies [32,37].

To ensure adequate vitamin D status, its supply from the diet is equally important, especially since Poland is located in Central Europe, where sunshine levels are relatively low from October to March [49]. On the other hand, diet as an alternative source of vitamin D for humans is, under Polish conditions, a less effective source than skin synthesis. A balanced diet, according to estimates, covers a maximum of 20% of daily vitamin D requirements [32]. In the absence or under minimal UVB-induced biosynthesis of vitamin D, the recommended dietary intake of vitamin D is 15 µg per day (600 IU) in Poland [43] to ensure optimal serum 25(OH)D level [32]. Therefore, strategies undertaken to improve vitamin D status in adolescents should include dietary recommendations. In order to formulate them, information on habitual vitamin D intake and its sources in the diet is necessary. The number of studies performed on this topic in the adolescent population is inadequate and has mostly involved small groups or selected regions of Poland. To our knowledge, this is the first study conducted among female post-primary school students representing all regions and voivodeships in Poland, in which a validated FFQ questionnaire was used to assess vitamin D intake [40].

Overall, vitamin D intake from food in the population-based cohort of Polish female sample adolescents was very low, representing only 15.5% of the recommendations. A low, but higher than in our study, vitamin D intake in a sample of Polish women aged 15–30 years was also observed by Utri and Głąbska [50], who used the same FFQ questionnaire to assess vitamin D intake [40]. Other studies conducted in Poland on groups of young women also obtained similar results [51,52,53,54,55].

An insufficient intake of vitamin D from food and beverages among adolescents is widely reported in Poland and other European countries. In two other studies in Polish girls aged 11–17 and 15–18 years [56,57], which used 24-h recalls to assess vitamin D intake, the mean intake from diet only was 3.2 and 3.0 µg per day, respectively, just slightly higher than that recorded in the current study. Similarly, in another study among adolescents aged 14–18 years using a semi-quantitative food frequency questionnaire (sq-FFQ) to assess vitamin D intake, the intake in Poland was only 2.9 µg per day [58]. Compared with various studies on the adolescent female population across the European countries, the intake of vitamin D in our study was lower than intake in Norway, Italy, the Netherlands, and Hungary and higher than intake in Austria, Spain, United Kingdom, Slovenia and Switzerland [29,31,33,48,58,59,60]. In general, adolescents from Southern and Northern European countries had higher vitamin D intakes than adolescents from Western Europe, while the lowest intakes were observed in Central and Eastern European countries [28].

The present study indicates that the problem of inadequate dietary vitamin D intake is very serious in the population of Polish female adolescents: more than 98% of the participants had a vitamin D intake lower than the recommended 15 µg and about 96% had a vitamin D intake lower than the Estimated Average Requirement (EAR, i.e., 10 µg), while 54% had a vitamin D intake lower than 2.5 µg, below the lower limit of reference intake value (LRNI) [29]. In studies by Głąbska et al. [40], Utri and Głąbska [50], Ponder et al. [51], and Przysławski et al. [55], 97%, 95%, 77%, and 71% of female respondents, respectively, did not reach the vitamin D intake equal to that recommended EAR. Compared to Polish female adolescents participating in our study, Slovenian female adolescents were more likely to have intakes below LRNI (about 73%) [59].

Natural sources of vitamin D in the Polish diet are limited and include products such as fish (eel, wild salmon, and herring) and fish products, being the richest source, and egg yolk, milk, and some mushrooms to a lesser extent [43]. The reduced choice of products that are sources of significant amounts of vitamin D and the low consumption of fish and fish dishes, therefore, results in an inadequate supply of vitamin D and a vitamin D deficiency, described as a pandemic in many European countries [28,32]. On the other hand, a meta-analysis of randomized controlled trials showed that fish consumption, as the main source of vitamin D in the diet, increases blood 25(OH)D concentrations [61]. It was reported that Scandinavian countries generally show higher values for circulating 25(OH)D than Southern Europe. This may be due to the consumption of more oil-rich fish, and greater use of cod liver oil and other vitamin D supplements [29]. In Eastern Europe, the status of vitamin D is poorer than in Northern and Western Europe [31]. Admittedly, our study noted that the main dietary source of vitamin D was fish and fish products, but they provided only 0.52 μg of vitamin D per day, which is insufficient to meet the recommended intake. In contrast, other foods providing vitamin D consumed by the female adolescents surveyed were meat and meat products, eggs, dairy products, cereal products, and fats.

In our study, participants’ total vitamin D intake was low, which was mainly due to low intake of its main source, namely fish and fish products. Participants did not follow the recommendations for a daily intake of two servings of fish [62]. Similar results have been obtained in studies assessing the dietary behavior or lifestyle of Polish adolescents [63,64,65]. In addition, in a study evaluating fish consumption in five European countries, including Poland, a minimum of one serving of fish per week was consumed by about 50% of Poles, Belgians, and Danes, as well as about 40% of Dutch and as many as nearly 90% of Spaniards [66].

At the same time, it should be emphasized that fish and fish products accounted for the largest share of daily vitamin D intake in our study, which is due to the consumption of the largest number of servings of this product group, as well as the consumption of fish with the highest vitamin D content. This finding is confirmed by the strong positive correlations recorded between the number of servings of fish, fish products, and fish with the highest vitamin D content, as well as the intake of vitamin D from them, and the total dietary intake of vitamin D. It is also noteworthy that in the 25% of adolescents studied who consumed no less than 4.03 µg of vitamin D (the third quartile of intake), the proportion of fish, especially those with the highest vitamin D content, was the highest in vitamin D supply (the fourth quartile of vitamin D intake). In France, Switzerland, and Spain, fish is also the main source of vitamin D in children and adolescents [39,60].

Fish species with the highest vitamin D content (salmon, rainbow trout, herring, and eel) [43] provided an average of 0.25 g per day. Salmon and herring were the most frequently chosen fish by the female students surveyed, and they were the source of the highest amount of vitamin D in the diet. This was particularly evident in those consuming vitamin D in the fourth quartile range (4.04–29.34 µg of vitamin D). In the other quartiles of vitamin D intake, the participation of the listed fish species as a source of vitamin D was increasingly smaller, reaching the smallest range in the subgroup in the first quartile. A comparable intake of vitamin D from fish with the highest vitamin D content was reported in a population-based sample of young Polish women [50].

One likely reason for low fish consumption, and not investigated in this study, is the price [67]; this is because the price has been shown to be a limiting factor in fish consumption in Poland [68]. Other equally important factors contributing to low fish consumption include the low level of public awareness of the importance of fish in daily nutrition, the limited availability and low species diversity of fresh fish and fish products, the instability of the raw material, problematic pre-processing (i.e., gutting, filleting), limited culinary skills in preparing fish dishes, fear of the presence of undesirable substances in fish meat, and aversion to the characteristic smell and taste of fish dishes and the presence of bones [69,70,71].

Variations in vitamin D intake in different countries are due not only to limited consumption of natural sources but also to different policies and strategies to prevent vitamin D deficiency at the population level. Some countries have introduced mandatory fortification of selected foods, such as milk, dairy, cereal, orange juice, margarine, and pasta. However, the approach to fortification varies from one region of the world to another. In Poland, food fortification has not been customary (globally or locally) to date, with the exception of milk formulas for infants and young children [34,35].

However, it is worth remembering that an important risk factor for vitamin D deficiency is also insufficient cutaneous vitamin D synthesis [28]. The present study was conducted during the winter-spring period, a period in which cutaneous vitamin D synthesis is virtually ineffective [49].

In addition to the amount and species of fish consumed, a factor that influenced the dietary intake of vitamin D in female post-primary school students included the region of Poland. The total consumption of this vitamin by participants attending schools in the Northern region was higher than that of those attending schools in the Eastern region. At the same time, participants attending schools in the Southern region consumed more vitamin D from fish and fish products than those attending schools in the Northern region. These results are relatively surprising, as there is usually a territorial variation in diet, as well as the very awareness of healthy food choices [65,72,73,74]. Moreover, different regions of Poland differ in the availability of fish and fish products—regions located in the North of Poland (close to the Baltic Sea and lakes) have much greater access compared to regions in the South. At the same time, to our knowledge, there are no studies comparing the consumption of fish and fish products in different regions of Poland.

In our study, almost half of the subjects declared taking vitamin D supplements. At the same time, the total intake of this vitamin from food was higher in girls declaring taking these supplements compared to those who declared not using vitamin D supplementation. It should be noted that this intake was still too low in relation to the recommendations, and this may have been one of the reasons for the implementation of supplements. The prevalence of the use of dietary supplements in the study group is consistent with the results of other studies [59,75,76,77]. At the same time, other studies have also shown that the intake of vitamin D from food among those using supplements (including those containing vitamin D) was insufficient [75,76,77,78].

Plant-based, i.e., vegetarian (excluding meat and fish) or vegan (exclusively plant-based foods) diets have become more popular in Western societies during the last decades, including among children and adolescents. A vegetarian diet is not only a trend but, rather, a lifestyle concept. Adolescents can use vegetarianism as a way of establishing identity, expressing value, and control of their lives. A vegetarian diet is associated with both benefits and health risks. This also applies to vitamin D intake because this diet eliminates the rich sources of this vitamin, i.e., fish. Unfortunately, data on food and nutrient intake in this group are lacking [79,80]. In various studies, vitamin D status and consumption did not differ between groups using a vegetarian diet and those not using it, or it was worse [81,82]. In our study, 8% of participants declared that they followed a vegetarian or vegan diet, while their vitamin D intake was lower than that of participants who did not follow any of these diets. It is therefore advisable to use diets that exclude meat and fish and have the consumption of all nutrients under control in order to prevent the development of deficiencies.

Given the low intake of vitamin D and fish by Polish female adolescents found in this study, in order to increase the supply of vitamin D and improve its status, a recommendation to increase the consumption of salmon and herring may be considered. This is because these fish are sources of significant amounts of vitamin D and were the most frequently chosen species of fish by female participants in the study.

## 5. Strengths and Limitations of the Study

In most of the studies mentioned in the discussion and finding low vitamin D intake, data on vitamin D consumption were collected by various methods: the 24-h dietary recall, dietary records (3 to 7 days), various types of FFQs, or both 24-h dietary recall and FFQs. In addition, most studies were conducted on small, heterogeneous, and unrepresentative samples. In contrast, our study was conducted on a large sample of young women from all regions of Poland and stands out for its representativeness and homogeneity. In addition, the results provided valuable data on vitamin D intake and its determinants in the population of Polish female adolescents. This is particularly important when developing dietary strategies to increase vitamin supply to recommended levels, and the FFQ used is characterized by a high relevance and a positive assessment of its validity and reproducibility [50]

The current study is limited by the fact that serum vitamin D levels were not determined, neither sunlight exposure (or exposure to artificial UV radiation) nor the dose of vitamin D supplements were estimated. As a result, the relationship between vitamin D intake and serum vitamin D levels and UV exposure, and vitamin D intake from supplements in the study sample is unknown. Unfortunately, this study was conducted only on the Polish population, so it is not known how the associations are shown in our study look in other countries.

## 6. Conclusions

In conclusion, dietary intake of vitamin D in the studied cohort of Polish female adolescents was alarmingly low, which was a consequence of its limited supply primarily from fish and fish products. The determinants of dietary vitamin D intake by Polish female adolescents were the amount and species of fish consumed, region of residence, use of vitamin D supplements, and following a vegetarian or vegan diet. In contrast, vitamin D intake was not associated with body mass index and age. The present study indicates an urgent need to develop strategies to increase the knowledge about the nutritional value and the health importance of vitamin D and to increase the intake of fish among this population.

## Figures and Tables

**Table 1:** Characteristics of the study group (according to the quartiles of total vitamin D intake from food).

Variable	Number (%) of Participants					*p* Value *
	Total	Quartiles of Vitamin D Intake from Food (µg/day)				
		Q1 (0.01–1.32)	Q2 (1.33–2.32)	Q3 (2.33–4.03)	Q4 (4.04–29.34)	
Total	4469 (100.00)	1117 (24.99)	1116 (24.97)	1118 (25.02)	1118 (25.02)	
Region of Poland:						
Central	567 (12.67)	149 (13.34)	132 (11.83)	144 (12.88)	142 (12.70)	0.00042
Eastern	914 (20.45)	283 (25.34)	197 (17.65)	220 (19.68)	214 (19.14)	
Northern	1350 (30.21)	299 (26.77)	342 (30.64)	325 (29.07)	384 (34.35)	
North-Western	396 (8.86)	99 (8.86)	110 (9.86)	99 (8.86)	88 (7.87)	
Southern	993 (22.22)	228 (20.41)	266 (23.83)	268 (23.97)	231 (20.66)	
South-Western	249 (5.57)	59 (5.18)	69 (6.18)	62 (5.54)	59 (5.28)	
Age:						0.14204
14–17	3131 (70.06)	812 (72.69)	764 (68.46)	768 (68.69)	787 (70.39)	
18–20	1338 (29.94)	305 (27.31)	352 (31.54)	350 (31.31)	331 (29.61)	
Body Mass Index (BMI) classification:						
Underweight	445 (9.96)	122 (10.92)	108 (9.768)	110 (9.84)	105 (9.39)	0.90188
Normal weight	3234 (72.36)	796 (71.26)	816 (73.12)	805 (72.00)	817 (73.08)	
Overweight	927 (10.78)	119 (10.65)	125 (11.20)	119 (10.64)	119 (10.64)	
Obesity	308 (6.89)	80 (7.16)	67 (6.00)	84 (7.51)	77 (6.88)	
Vitamin D supplementation:						
No	2222 (49.72)	630 (56.40)	555 (49.73)	508 (45.44)	529 (47.32)	<0.00001
Yes	2247 (50.28)	487 (43.60)	561 (50.27)	610 (54.56)	589 (52.68)	
Vegetarian or vegan diet:						
No	4113 (92.03)	964 (86.30)	1051 (94.18)	1046 (93.56)	1052 (94.10)	<0.00001
Yes	356 (7.97)	153 (3.70)	65 (5.82)	72 (6.44)	66 (5.90)	

* Chi-squared test for comparisons of proportions, *p* ≤ 0.05.

**Table 2:** Vitamin D intake from various food groups in the group of female post-primary school students (according to the quartiles of total vitamin D intake from food).

Vitamin D Source	Median (Min–Max) ** in µg/day					*p* Value ***
	Total	Quartiles of Vitamin D Intake from Food (µg/day)				
		Q1	Q2	Q3	Q4	
Vitamin intake from all food sources	2.33 (0.01–29.34)	0.87 (0.01–1.32) ^A^	1.81 (1.33–2.32) ^B^	3.00 (2.33–4.03) ^C^	5.96 (4.04–29.34) ^D^	<0.001
Intake from fish and fish products	0.52 (0.00–27.28)	0.00 (0.00–1.18) ^A^	0.27 (0.00–2.30) ^B^	1.18 (0.00–3.92) ^C^	3.32 (0.00–27.28) ^D^	<0.001
Salmon, rainbow trout, herring, eel	0.25 (0.00–21.25)	0.00 (0.00–1.07) ^A^	0.00 (0.00–1.88) ^B^	0.63 (0.00–3.17) ^C^	2.24 (0.00–21.25) ^D^	<0.001
• Salmon	0.23 (0.00–9.5)	0.00 (0.00–0.95) ^A^	0.00 (0.00–1.58) ^B^	0.00 (0.00–3.17) ^C^	0.63 (0.00–9.5) ^D^	<0.001
• Herring	0.18 (0.00–7.5)	0.00 (0.00–0.75) ^A^	0.00 (0.00–1.25) ^B^	0.00 (0.00–3.00) ^C^	0.25 (0.00–7.5) ^D^	<0.001
Halibut, mackerel, brook trout, sole, tuna	0.00 (0.00–5.88)	0.00 (0.00–0.67) ^A^	0.00 (0.00–1.33) ^B^	0.08 (0.00–3.33) ^C^	0.33 (0.00–5.88) ^D^	<0.001
Cod, flounder, plaice, pollock, hake	0.00 (0.00–1.25)	0.00 (0.00–0.50) ^A^	0.00 (0.00–0.50) ^B^	0.00 (0.00–0.87) ^C^	0.03 (0.00–1.25) ^D^	<0.001
Herring, sardine, and tuna products	0.00 (0.00–12.36)	0.00 (0.00–0.41) ^A^	0.00 (0.00–1.24) ^B^	0.00 (0.00–1.65) ^C^	0.41 (0.00–12.36) ^D^	<0.001
Other fish products	0.00 (0.00–0.93)	0.00 (0.00–0.62) ^A^	0.00 (0.00–0.31) ^B^	0.00 (0.00–0.31) ^C^	0.00 (0.00–0.93) ^D^	<0.001
Dairy products	0.31 (0.00–4.93)	0.19 (0.00–1.04) ^A^	0.30 (0.00–2.29) ^B^	0.36 (0.00–2.05) ^C^	0.50 (0.00–4.93) ^D^	<0.001
Eggs	0.38 (0.00–8.71)	0.12 (0.00–1.21) ^A^	0.37 (0.00–1.71) ^B^	0.50 (0.00–2.94) ^C^	0.75 (0.00–8.71) ^D^	<0.001
Meat and meat products	0.39 (0.00–4.33)	0.13 (0.00–1.14) ^A^	0.39 (0.00–2.00) ^B^	0.48 (0.00–2.83) ^C^	0.68 (0.00–4.33) ^D^	<0.001
Cereals	0.12 (0.00–2.48)	0.07 (0.00–0.86) ^A^	0.11 (0.00–0.86) ^B^	0.14 (0.00–2.04) ^C^	0.17 (0.00–2.48) ^D^	<0.001
Fats	0.04 (0.00–3.20)	0.01 (0.00–0.48) ^A^	0.04 (0.00–1.33) ^B^	0.05 (0.00–1.58) ^C^	0.07 (0.00–3.20) ^D^	<0.001

** Non-parametric distribution (verified using Shapiro-Wilk test, *p* ≤ 0.05), *** compared using Kruskal-Wallis analysis of variance (ANOVA), values with different letters (A, B, C, D) in rows differ significantly, *p* ≤ 0.05.

**Table 3:** The declared number of fish and fish product servings consumed per week in the group of female post-primary school students (according to the quartiles of total vitamin D intake from food).

Vitamin D Source	Median (Min–Max) ** in Servings/Week					*p* Value ***
	Total	Quartiles of Vitamin D Intake from Food (µg/day)				
		Q1	Q2	Q3	Q4	
Total fish and fish products	0.70 (0.00–34.53)	0.00 (0.00–7.00) ^A^	0.47 (0.00–7.00) ^B^	1.40 (0.00–12.83) ^C^	3.73 (0.00–34.53) ^D^	<0.001
Salmon, rainbow trout, herring, eel	0.23 (0.00–14.00)	0.00 (0.00–0.93) ^A^	0.00 (0.00–1.52) ^B^	0.47 (0.00–2.80) ^C^	1.63 (0.00–14.00) ^D^	<0.001
• Salmon	0.23 (0.00–7.00)	0.00 (0.00–0.70) ^A^	0.00 (0.00–1.17) ^B^	0.00 (0.00–2.80) ^C^	0.47 (0.00–7.00) ^D^	<0.001
• Herring	0.21 (0.00–7.00)	0.00 (0.00–0.70) ^A^	0.00 (0.00–1.17) ^B^	0.00 (0.00–2.33) ^C^	0.45 (0.00–7.00) ^D^	<0.001
Halibut, mackerel, brook trout, sole, tuna	0.00 (0.00–12.13)	0.00 (0.00–2.10) ^A^	0.00 (0.00–2.33) ^B^	0.23 (0.00–5.8) ^C^	0.70 (0.00–12.13) ^D^	<0.001
Cod, flounder, plaice, pollock, hake	0.00 (0.00–19.13)	0.00 (0.00–7.00) ^A^	0.00 (0.00–7.00) ^B^	0.00 (0.00–12.83) ^C^	0.47 (0.00–19.13) ^D^	<0.001
Herring, sardine, and tuna products	0.00 (0.00–7.00)	0.00 (0.00–0.23) ^A^	0.00 (0.00–0.70) ^B^	0.00 (0.00–0.93) ^C^	0.23 (0.00–7.00) ^D^	<0.001
Other fish products	0.00 (0.00–7.00)	0.00 (0.00–4.47) ^A^	0.00 (0.00–2.33) ^B^	0.00 (0.00–2.33) ^C^	0.00 (0.00–7.00) ^D^	<0.001

** Non-parametric distribution (verified using Shapiro-Wilk test, *p* ≤ 0.05), *** compared using Kruskal–Wallis analysis of variance (ANOVA), values with different letters (A, B, C, D) in rows differ significantly, *p* ≤ 0.05.

**Table 4:** Comparison of vitamin D intake from various food groups (µg per day) in the subgroups of female post-primary school students from different regions of Poland.

Vitamin D Source	Regions of Poland												*p* Value ***
	Central (*n* = 567)		Eastern (*n* = 914)		Northern (*n* = 1350)		North-Western (*n* = 396)		Southern (*n* = 993)		South-Western (*n* = 249)		
	Intake (%)	Median ** (Min–Max)	Intake (%)	Median ** (Min–Max)	Intake (%)	Median ** (Min–Max)	Intake (%)	Median ** (Min–Max)	Intake (%)	Median ** (Min–Max)	Intake (%)	Median ** (Min–Max)	
Total intake from food	100.00	2.35 (0.01–25.18)	100.00	2.20 ^A^ (0.01–29.34)	100.00	2.41 ^B^ (0.04–29.26)	100.00	2.22 (0.05–28.17)	100.00	2.33 (0.07–24.27)	100.00	2.24 (0.13–23.68)	0.0008
Intake from fish and fish products Salmon, rainbow trout, herring, and eel Halibut, mackerel, brook trout, sole, and tuna Cod, flounder, plaice, pollock, and hake Herring, sardine, and tuna products Other fish products	32.08	0.60 (0.00–21.10) 0.26 (0.00–18.47) 0.03 ^AC^ (0.00–5.60) 0.00 (0.00–1.25) 0.00 (0.00–8.24) 0.00 (0.00–0.62)	34.33	0.58 (0.00–17.89) 0.25 (0.00–12.95) 0.03 ^AC^ (0.00–5.32) 0.00 (0.00–0.50) 0.00 (0.00–12.36) 0.00 (0.00–0.93)	27.87	0.43 ^A^ (0.00–27.28) 0.25 (0.00–21.25) 0.00 ^BC^ (0.00–5.88) 0.00 (0.00–0.78) 0.00 ^A^ (0.00–6.18) 0.00 (0.00–0.62)	29.93	0.49 (0.00–21.47) 0.25 (0.00–21.19) 0.00 ^C^ (0.00–2.56) 0.00 (0.00–1.14) 0.00 (0.00–8.24) 0.00 (0.00–0.31)	32.01	0.63 ^B^ (0.00–18.39) 0.25 (0.00–13.31) 0.08 ^A^ (0.00–5.42) 0.00 (0.00–0.89) 0.00 ^B^ (0.00–8.24) 0.00 (0.00–0.31)	28.83	0.43 (0.00–19.90) 0.25 (0.00–17.00) 0.00 (0.00–4.00) 0.00 (0.00–0.62) 0.00 (0.00–3.30) 0.00 (0.00–0.25)	0.0329 0.6626 <0.0001 0.102 <0.0001 0.4128
Dairy products Eggs Meat and meat products Cereals Fats	16.39 23.12 18.07 6.93 3.41	0.31 (0.00–3.29) 0.37 ^A^ (0.00–7.43) 0.36 ^A^ (0.00–3.47) 0.11 ^A^ (0.00–2.04) 0.03 ^ACD^ (0.00–2.03)	23.82 16.56 15.64 6.25 3.40	0.31 (0.00–3.84) 0.25 ^B^ (0.00–7.50) 0.30 ^B^ (0.00–3.94) 0.10 ^B^ (0.00–2.48) 0.03 ^B^ (0.00–2.11)	17.02 22.00 22.42 6.68 4.00	0.32 (0.00–4.93) 0.49 ^A^ (0.00–8.71) 0.47 ^C^ (0.00–4.33) 0.12 ^A^ (0.00–1.99) 0.04 ^C^ (0.00–2.88)	18.17 21.18 20.13 7.60 2.99	0.34 (0.00–3.13) 0.49 ^A^ (0.00–5.00) 0.43 ^AC^ (0.00–3.60) 0.12 ^A^ (0.00–1.82) 0.03 ^CD^ (0.00–1.86)	16.60 21.16 18.66 7.21 4.37	0.30 (0.00–3.16) 0.49 ^A^ (0.00–6.79) 0.37 ^A^ (0.00–4.01) 0.12 ^A^ (0.00–1.06) 0.05 ^BD^ (0.00–3.20)	17.54 21.34 20.81 7.30 4.18	0.32 (0.00–2.30) 0.49 ^A^ (0.00–6.29) 0.45 ^AC^ (0.00–3.6) 0.12 ^A^ (0.00–1.69) 0.05 ^BD^ (0.00–2.93)	0.19 <0.0001 <0.0001 <0.0001 <0.0001

** Non-parametric distribution (verified using Shapiro-Wilk test, *p* ≤ 0.05), *** compared using Kruskal-Wallis analysis of variance (ANOVA), values with different letters (A, B, C, D) in rows differ significantly, *p* ≤ 0.05.

**Table 5:** Comparison of dietary vitamin D intake from various food groups (µg per day) in the age subgroups of female post-primary school students.

Vitamin D Source	Age of Participants				*p* Value ***
	14–17 Years (*n* = 3131)		18–20 Years (*n* = 1338)		
	Intake (%)	Median ** (Min–Max)	Intake (%)	Median ** (Min–Max)	
Total intake from food	100.00	2.36 (0.02–29.34)	100.00	2.41 (0.01–29.26)	0.2935
Intake from fish and fish products Salmon, rainbow trout, herring, and eel Halibut, mackerel, brook trout, sole, and tuna Cod, flounder, plaice, pollock, and hake Herring, sardine, and tuna products Other fish products	29.31	0.50 (0.00–27.23) 0.25 (0.00–21.25) 0.00 (0.00–5.60) 0.00 (0.00–1.25) 0.00 (0.00–12.36) 0.00 (0.00–0.93)	30.54	0.61 (0.00–27.28) 0.26 (0.00–20.93) 0.00 (0.00–5.88) 0.00 (0.00–0.56) 0.00 (0.00–8.24) 0.00 (0.00–0.62)	0.1038 0.0800 06081 0.3913 0.3335 0.1275
Dairy products Eggs Meat and meat products Cereals Fats	17.61 21.52 20.54 7.14 3.88	0.32 (0.00–3.84) 0.49 (0.00–8.71) 0.39 (0.00–4.20) 0.12 (0.00–2.04) 0.04 (0.00–3.20)	16.61 21.98 19.46 7.22 4.19	0.31 (0.00–4.93) 0.49 (0.00–7.43) 0.43 (0.00–4.33) 0.13 (0.00–2.48) 0.05 (0.00–2.68)	0.1091 0.1778 0.7289 0.0081 0.0121

** Non-parametric distribution (verified using Shapiro-Wilk test, *p* ≤ 0.05), *** verified using Mann-Whitney U test, *p* ≤ 0.05.

**Table 6:** Comparison of vitamin D intake from various food groups (µg per day) in the sub-groups of underweight, normal weight, overweight and obese female post-primary school students.

Vitamin D Source	Body Mass Index (BMI) Classification								*p* Value ***
	Underweight (*n* = 445)		Normal Weight (*n* = 3234)		Overweight (*n* = 482)		Obesity (*n* = 308)		
	Intake (%)	Median ** (Min–Max)	Intake (%)	Median ** (Min–Max)	Intake (%)	Median ** (Min–Max)	Intake (%)	Median ** (Min–Max)	
Total intake from food	100.00	2.24 (0.01–23.14)	100.00	2.33 (0.02–29.3)	100.00	2.30 (0.04–24.98)	100.00	2.42 (0.01–25.97)	0.7471
Intake from fish and fish products Salmon, rainbow trout, herring, and eel Halibut, mackerel, brook trout, sole, and tuna Cod, flounder, plaice, pollock, and hake Herring, sardine, and tuna products Other fish products	29.53	0.46 (0.00–19.18) 0.25 (0.00–15.20) 0.00 (0.00–3.20) 0.00 (0.00–0.56) 0.00 (0.00–8.24) 0.00 (0.00–0.37)	31.04	0.53 (0.00–27.28) 0.25 (0.00–21.25) 0.00 (0.00–5.90) 0.00 (0.00–1.25) 0.00 (0.00–12.36) 0.00 (0.00–0.93)	30.71	0.50 (0.00–18.79) 0.25 (0.00–17.50) 0.00 (0.00–4.00) 0.00 (0.00–0.63) 0.00 (0.00–4.94) 0.00 (0.00–0.62)	31.45	0.47 (0.00–21.47) 0.25 (0.00–15.53) 0.00 (0.00–4.20) 0.00 (0.00–0.80) 0.00 (0.00–0.62) 0.00 (0.00–0.31)	0.7428 0.723 0.7571 0.5637 0.7054 0.7914
Dairy products Eggs Meat and meat products Cereals Fats	18.48 20.78 19.80 7.10 4.32	0.30 (0.00–3.84) 0.36 (0.00–8.71) 0.40 (0.00–3.60) 0.12 (0.00–0.98) 0.04 (0.00–1.01)	18.43 20.67 19.07 6.98 3.82	0.32 (0.00–4.93) 0.38 (0.00–8.11) 0.37 (0.00–4.20) 0.12 (0.00–2.48) 0.04 (0.00–3.20)	17.56 21.62 20.43 6.04 3.63	0.30 (0.00–3.16) 0.49 (0.00–7.50) 0.40 (0.00–4.09) 0.14 (0.00–0.98) 0.04 (0.00–2.93)	18.82 19.94 20.05 6.53 3.20	0.31 (0.00–3.33) 0.43 (0.00–3.71) 0.42 (0.00–4.33) 0.10 (0.00–2.04) 0.03 (0.00–1.85)	0.1302 0.5984 0.7790 0.364 0.1844

** Non-parametric distribution (verified using Shapiro-Wilk test, *p* ≤ 0.05), *** compared using Kruskal-Wallis analysis of variance (ANOVA).

**Table 7:** Comparison of vitamin D intake from various food groups (µg per day) in the subgroups of female post-primary school students not supplementing and supplementing vitamin D, and following omnivorous vs. vegetarian or vegan diet.

Vitamin D Source	Vitamin D Supplementation					Vegetarian or Vegan Diet				
	No (*n* = 2222)		Yes (*n* = 2247)		*p* Value ***	No (*n* = 4113)		Yes (*n* = 356)		*p* Value ***
	Intake (%)	Median ** (Min-Max)	Intake (%)	Median ** (Min–Max)		Intake (%)	Median ** (Min–Max)	Intake (%)	Median ** (Min–Max)	
Total intake from food	100.00	2.19 (0.02–28.17)	100.00	2.47 (0.01–29.34)	<0.0001	100.00	2.37 (0.01–29.34)	100.00	1.70 (0.01–28.75)	<0.0001
Intake from fish and fish products Salmon, rainbow trout, herring, and eel Halibut, mackerel, brook trout, sole, and tuna Cod, flounder, plaice, pollock, and hake Herring, sardine, and tuna products Other fish products	29.03	0.42 (0.00–27.22) 0.25 (0.00–21.25) 0.00 (0.00–5.60) 0.00 (0.00–1.25) 0.00 (0.00–12.36) 0.00 (0.00–0.93)	32.72	0.61 (0.00–27.28) 0.32 (0.00–21.19) 0.08 (0.00–5.88) 0.00 (0.00–1.14) 0.00 (0.00–8.24) 0.00 (0.00–0.93)	<0.0001 0.0001 <0.0001 0.0002 0.0647 0.1163	31.33	0.55 (0.00–27.28) 0.00 (0.00–21.25) 0.00 (0.00–5.88) 0.00 (0.00–1.25) 0.00 (0.00–12.36) 0.00 (0.00–0.93)	28.20	0.09 (0.00–19.66) 0.00 (0.00–13.76) 0.00 (0.00–2.42) 0.00 (0.00–0.50) 0.00 (0.00–8.24) 0.00 (0.00–0.31)	<0.0001 0.0002 0.0001 0.0174 0.0059 0.0015
Dairy products Eggs Meat and meat products Cereals Fats	18.77 20.58 20.64 7.02 3.95	0.30 (0.00–4.93) 0.37 (0.00–7.43) 0.39 (0.00–4.33) 0.11 (0.00–1.99) 0.04 (0.00–3.20)	17.97 20.88 18.08 6.69 3.67	0.33 (0.00–3.84) 0.49 (0.00–8.71) 0.38 (0.00–4.09) 0.12 (0.00–2.48) 0.04 (0.00–2.93)	0.0002 0.0026 0.2341 0.1837 0.8308	17.68 20.30 20.31 6.65 3.72	0.31 (0.00–4.93) 0.38 (0.00–8.12) 0.42 (0.00–4.33) 0.12 (0.00–2.48) 0.04 (0.00–3.20)	28.41 28.19 0.00 10.09 5.11	0.29 (0.00–2.00) 0.36 (0.00–8.71) 0.00 (0.00–0.00) 0.11 (0.00–1.33) 0.03 (0.00–2.30)	0.5086 0.206 <0.0001 0.0889 0.0007

** Non-parametric distribution (verified using Shapiro-Wilk test, *p* ≤ 0.05), *** verified using Mann-Whitney U test, *p* ≤ 0.05.

**Table 8:** The correlations between the declared number of servings of fish and fish products per week and vitamin D intake from them, and BMI and total dietary vitamin D intake in the group of female post-primary school students.

Variable		Correlations	
		r	*p*
Number of servings of fish and fish products per week	Total fish and fish products	0.74	<0.01
	Salmon, rainbow trout, herring, and eel	0.71	<0.01
	Halibut, mackerel, brook trout, sole, and tuna	0.56	<0.01
	Cod, flounder, plaice, pollock, and hake	0.43	<0.01
	Herring, sardine, and tuna products	0.54	<0.01
	Other fish products	0.41	<0.01
Vitamin D intake from fish and fish products (µg/day)	Intake from fish and fish products	0.76	<0.01
	Salmon, rainbow trout, herring, and eel	0.71	<0.01
	Halibut, mackerel, brook trout, sole, and tuna	0.56	<0.01
	Cod, flounder, plaice, pollock, and hake	0.43	<0.01
	Herring, sardine, and tuna products	0.54	<0.01
	Other fish products	0.42	<0.01
Body Mass Index (kg/m^2^)		0.03	0.0093

## Data Availability

Not applicable.
